# Optimizing and Individualizing the Pharmacological Treatment of First-Episode Schizophrenic Patients: Study Protocol for a Multicenter Clinical Trial

**DOI:** 10.3389/fpsyt.2021.611070

**Published:** 2021-02-25

**Authors:** Jingmei Xiao, Jing Huang, Yujun Long, Xiaoyi Wang, Ying Wang, Ye Yang, Gangrui Hei, Mengxi Sun, Jin Zhao, Li Li, Tiannan Shao, Weiyan Wang, Dongyu Kang, Chenchen Liu, Peng Xie, Yuyan Huang, Renrong Wu, Jingping Zhao

**Affiliations:** Department of Psychaitry, National Clinical Research Center for Mental Disorders, The Second Xiangya Hospital of Central South University, Changsha, China

**Keywords:** first-episode schizophrenic patients, optimized and individualized treatment, metabolic syndrome, biomarker, efficacy and adverse effects

## Abstract

**Introduction:** Affecting ~1% of the world population, schizophrenia is known as one of the costliest and most burdensome diseases worldwide. Antipsychotic medications are the main treatment for schizophrenia to control psychotic symptoms and efficiently prevent new crises. However, due to poor compliance, 74% of patients with schizophrenia discontinue medication within 1.5 years, which severely affects recovery and prognosis. Through research on intra and interindividual variability based on a psychopathology–neuropsychology–neuroimage–genetics–physiology-biochemistry model, our main objective is to investigate an optimized and individualized antipsychotic-treatment regimen and precision treatment for first-episode schizophrenic patients.

**Methods and Analysis:** The study is performed in 20 representative hospitals in China. Three subprojects are included. In subproject 1, 1,800 first-episode patients with schizophrenia are randomized into six different antipsychotic monotherapy groups (olanzapine, risperidone, aripiprazole, ziprasidone, amisulpride, and haloperidol) for an 8-week treatment. By identifying a set of potential biomarkers associated with antipsychotic treatment response, we intend to build a prediction model, which includes neuroimaging, epigenetics, environmental stress, neurocognition, eye movement, electrophysiology, and neurological biochemistry indexes. In subproject 2, apart from verifying the prediction model established in subproject 1 based on an independent cohort of 1,800 first-episode patients with schizophrenia, we recruit patients from a verification cohort who did not get an effective response after an 8-week antipsychotic treatment into a randomized double-blind controlled trial with minocycline (200 mg per day) and sulforaphane (3 tables per day) to explore add-on treatment for patients with schizophrenia. Two hundred forty participants are anticipated to be enrolled for each group. In subproject 3, we tend to carry out one trial to construct an intervention strategy for metabolic syndrome induced by antipsychotic treatment and another one to build a prevention strategy for patients at a high risk of metabolic syndrome, which combines metformin and lifestyle intervention. Two hundred participants are anticipated to be enrolled for each group.

**Ethics and Dissemination:** The study protocol has been approved by the Medical Ethics committee of the Second Xiangya Hospital of Central South University (No. 2017027). Results will be disseminated in peer-reviewed journals and at international conferences.

**Trial Registration:** This trial has been registered on Clinicalrials.gov (NCT03451734). The protocol version is V.1.0 (April 23, 2017).

## Strengths and Limitations

- This multicenter trial will build a large bank for first-episode schizophrenic patients in a Chinese population which includes clinical data and biological samples.
- Biomarkers including social psychology, neuroimage, genetics, and biochemistry will be identified to build a prediction model for an optimized and individualized treatment.
- Add-on treatment and intervention strategy will be explored for those with bad treatment response and significant metabolic disturbance.
- Although randomized in subproject 1, participants will be informed about the detailed information of drugs, which may contribute to the imbalance between six groups.
- Participants in acute episode cannot cooperate with evaluations such as MRI examination and MCCB assessment, which leads to data loss and bias in statistical analysis.

## Introduction

**Figure 1:**
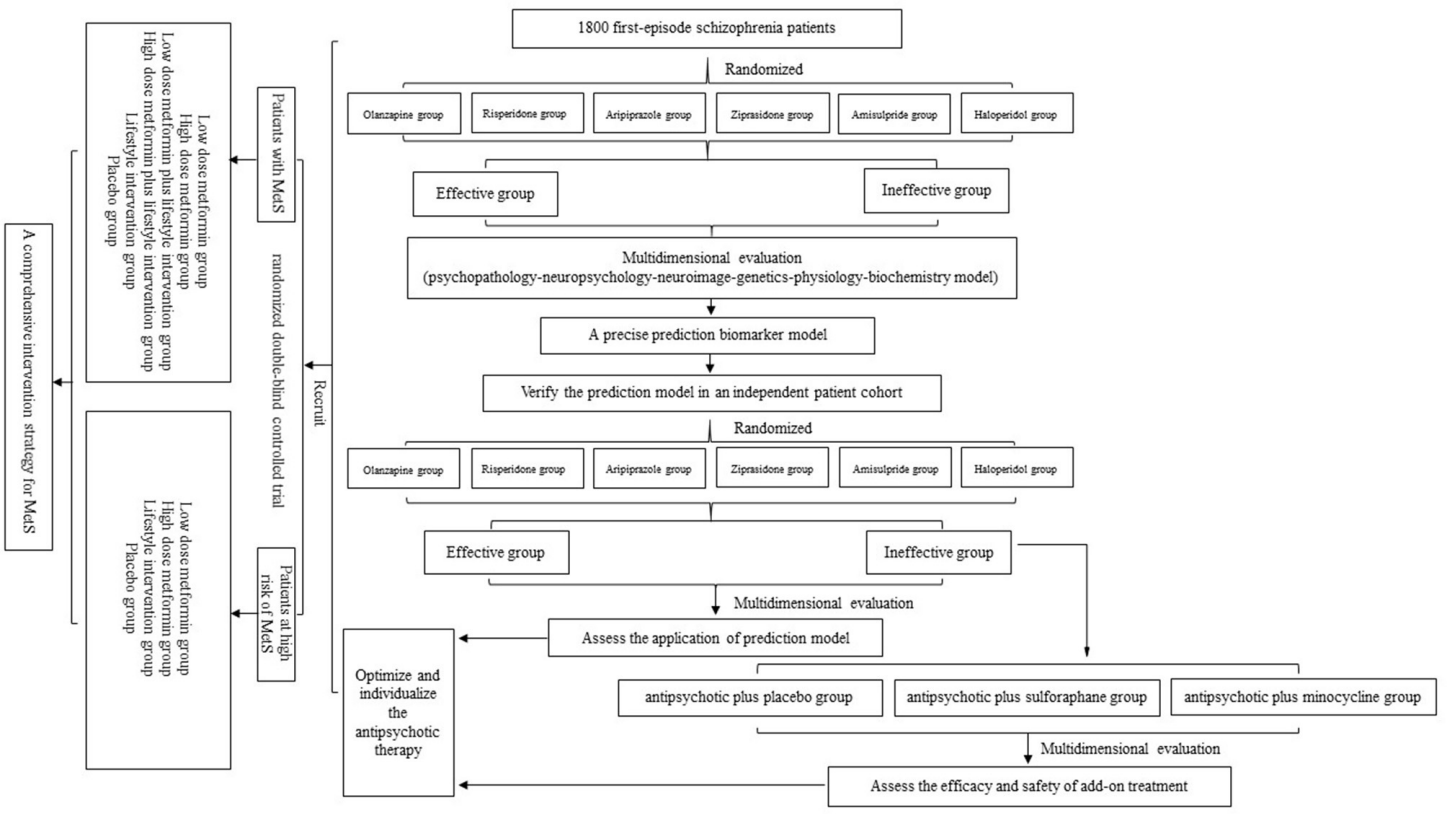
Study flow diagram. In subproject 1, after recruitment and eligibility checks, 1,800 participants will be randomized into six monotherapy groups. Through an 8-week multidimensional evaluation, we will screen potential biomarkers and build a prediction model. Then, the verification part and add-on treatment will be carried out in subproject 2. Finally, we will conduct a randomized double-blind controlled trial to summarize a comprehensive intervention strategy for metabolic syndrome (MetS).

Schizophrenia is a psychotic disorder that impairs self and social functioning. Affecting ~1% of the world population, it is also known as one of the costliest and most burdensome diseases worldwide (1). To date, antipsychotic medications are the main treatment for schizophrenia, and these drastically control psychotic symptoms and efficiently prevent new crises. However, due to poor compliance, 74% of patients with schizophrenia discontinue medication within 1.5 years (2), which severely affects their recovery and prognosis. More importantly, there are interindividual differences in responses to antipsychotic medications, in terms of either efficacy or safety. Approximately one-third of patients have no response to antipsychotics (3), and patients have diverse susceptibility to antipsychotic-induced adverse effects, such as extrapyramidal symptoms (EPS), tardive dyskinesia (TD), and metabolic syndrome (MetS) (4). Therefore, it is urgent to find biomarkers to predict the efficacy and side effects of antipsychotic treatment for first-episode schizophrenic patients. In this protocol, we describe the overall design of the project (Figure 1), the task assignment of three subprojects, and details of study evaluations and follow-up. Through research on intra- and interindividual variability based on the psychopathology–neuropsychology–neuroimaging–genetics–physiology–biochemistry model, our main objective is to investigate an optimized and individualized antipsychotic-treatment project and precision treatment for first-episode schizophrenic patients.

## Methods and Analysis

### Subproject 1

#### Aim

To establish a biomarker model to predict clinical outcomes for the six most commonly used antipsychotic medications (olanzapine, risperidone, aripiprazole, ziprasidone, amisulpride, and haloperidol).

#### Study Design

First, we build a clinical database system and a biological sample bank for data and sample management, which is applicable to other hospitals in this project. Specially, we use pmsampsize-type b with 0.2 rsquared to calculate the minimum sample size required to develop a multivariable prediction model for a binary outcome using 35 candidate predictor parameters. Assuming 0.05 acceptable difference in apparent and adjusted R-squared, 0.05 margin of error in estimation of intercept, and 0.39 prevalence related to events per predictor parameter (5), we get a minimum sample size with 20% of participants lost to follow-up which is 1,741. Therefore, 1,800 first-episode schizophrenic patients will be recruited at 19 sites and randomized into six treatment groups (olanzapine, risperidone, aripiprazole, ziprasidone, amisulpride, haloperidol). As the initiating hospital, the Second Xiangya Hospital of Central South University undertakes the task of enrolling and following up with 780 participants, the Xiangya Hospital of Central South University only performs analysis work, and the remaining 17 collaborative hospitals each enrolls and follows up 60 participants. We use a random number table to decide the antipsychotic monotherapy groups of participants, and we anticipate that every monotherapy group will have an equal distribution. Through an 8-week treatment and follow-up, we collect multidimensional indexes from psychopathology, neuropsychology, brain imaging, physiology, biochemistry, and life stress data. The summarized data will be analyzed to screen potential biomarkers or biomarker panel that might predict the antipsychotic response, to ultimately establish a prediction model.

#### Inclusion Criteria

Participants are eligible to enter the trial under the following conditions: (1) participants must be aged 18–65 years, meeting the criteria for schizophrenia diagnosed by Diagnostic and Statistical Manual of Mental Disorders—fifth edition (DSM-5) or International Classification of Diseases—tenth edition (ICD-10); (2) participants must be experiencing a current episode of psychotic symptoms with a disease course <3 years and intermission <6 months; (3) at least 1 guardian must be available to accompany the patient within 1 year; and (4) participants and their guardians must sign an informed consent form (ICF) indicating that they understand the purpose of and procedures required for the study and are willing to participate in the study.

#### Exclusion Criteria

Any potential participant who meets any of the following criteria will be excluded from participating in the study: (1) participant has a known or suspected clinically unstable systemic medical disorder; (2) participant has a current or prior DSM-5/ICD-10 diagnosis of substance use disorder, intellectual disability, autism spectrum disorder, dementia, or severe cognitive impairment; (3) participant is planning to be pregnant, is pregnant, or is breast-feeding; and (4) participant is currently enrolled in another clinical trial.

#### Procedures

For clinical and biological data collection, we collect blood samples and case information, which contains demographic data, medical history (including medicine history, personal history, and family history), medication regimen, and results of follow-up evaluation. The biological samples are stored using a unique code for which storage information can be linked to the research case in the web server.

The standard operation procedures (SOPs) are followed for data collection, storage, tracking, and utilization. Professional technicians are employed for the establishment and subsequent maintenance of the internet platform, including web server and mobile terminal applications (APP). Ancillary researchers are involved in the collection and timely uploading of case information online. Principal investigators are responsible for auditing the data and quality control. Only the program director, the highest authority, is able to check all case information from 19 hospitals.

The procedures on processing and storage of blood sample are unified as follows: we collect blood samples into four tubes when patients are in a fasting state from 7 to 9 am. Tube 1 (10-ml EDTA anticoagulation tube): the blood is centrifuged for 10 min (i.e., 2,000 × g, 4°C). Then, the separated plasma and hemocytes are, respectively, dispensed into three EP tubes (for backups and the measurement of cytokine and oxidative stress indexes) and two EP tubes (for the measurement of gene indexes) and finally transferred to a −80°C freezer for long-term storage.

Tube 2 (5-ml separation gel vacuum procoagulant tube): the blood is centrifuged for 10 min (i.e., 2,000 × g, 4°C), and then the separated serum is dispensed into three EP tubes (for backups and the measurement of metabolomics indexes) and transferred to a −80°C freezer for long-term storage.

Tube 3 (10-ml PAXgene blood RNA tube): the blood is stored in a −20°C freezer as soon as collected and transferred to a −80°C freezer after 24 h.

Tube 4 (10-ml EDTA anticoagulation tube): peripheral blood mononuclear cells (for measuring epigenetic indexes) are extracted from the blood sample within 4 h after collection; then, they are dispensed into EP tubes and transferred to a −80°C freezer for long-term storage. Each sample is stored using its own code for which storage information can be linked to the research case in the web server.

Participants are randomized into olanzapine, risperidone, aripiprazole, amisulpride, ziprasidone, and haloperidol groups. They are evaluated and followed at baseline, week 4, and week 8 with the following parameters: efficacy evaluation, neurocognitive assessment, safety assessment, measurement of eye movement, magnetic resonance imaging (MRI) examination, measurement of pharmacogenomics, epigenetic indexes, neuroimmunological indexes, metabolomics indexes, and metabolic signs. The details of every visit are described in Tables 1, 2.

**Table 1:** Details of follow-up (subproject 1 and 2).

**Follow-up**	**1 (baseline)**	**2 (4th week)**	**3 (8th week)**
Week	0	4	8
ICF	√		
Inclusive/exclusive criterion	√		
Demographic information	√		
Medical history	√		
Medication regimen and compliance	√	√	√
Physical examination	√	√	√
Auxiliary examination	√	√	√
PANSS	√	√	√
GAF	√	√	√
CGI	√	√	√
TESS		√	√
SAFTEE		√	√
RESES		√	√
BAS		√	√
Eye movement	√		√
MCCB	√		√
MRI	√		√
Collection of blood sample	√		√

**Table 2:** Details of laboratory test (subproject 1 and 2).

**Follow-up**	**1 (baseline)**	**2 (4th week)**	**3 (8th week)**
Week	0	4	8
Blood regulation	√	√	√
Liver function	√	√	√
Renal function	√	√	√
Blood lipids	√	√	√
FBG	√	√	√
HBV, HCV	√		
Thyroid function	√		

According to participants' response to treatment after an 8-week follow-up, they are assigned into four groups as follows: effectiveness and mild side-effect group, effectiveness and severe side-effect group, ineffectiveness and mild side-effect group, and ineffectiveness and severe side-effect group. Univariate analysis will be used to identify biomarkers associated with treatment efficacy and side effects, which will be used to establish a prediction model. Then, we will apply machine learning methods to build a model that includes multiple linear regression, a naive Bayesian classifier, Bayesian networks, artificial neural networks, or decision-making tree analysis, among others. We will finally choose the prediction model based on the easiest and simplest computation for clinical application.

### Subproject 2

#### Aim

The aim was to verify the prediction model established in subproject 1 with an independent patient cohort and evaluate the effectiveness and safety of minocycline and sulforaphane as an add-on treatment.

This project, as an extension of subproject 1, includes verification of the prediction model established in subproject 1 and optimization of the current therapy with add-on treatment. First, validation of the prediction model is performed with an independent patient cohort. Next, we apply the add-on treatment to the patients who do not have an ideal response to antipsychotic treatment after an 8-week treatment. According to these results, we will construct an optimized and individualized therapy regimen for schizophrenia.

##### To Verify the Efficacy of the Prediction Model With an Independent Patient Cohort

After confirming the strong correlation between biomarkers and clinical outcomes and further establishing a prediction model in subproject 1, it next is important to perform clinical validation processes.

There are 1,800 first-episode schizophrenia patients recruited from 19 hospitals, and six groups as with subproject 1. We apply the prediction model established previously in the independent patient cohort and determines the monotherapy group for patients according to the prediction results. The assessments (timepoint and content) are conducted as in subproject 1.

Similarly, according to each individual's response to antipsychotic treatment (effectiveness/ineffectiveness and mild/severe side effects), we assign patients who completed the entire 8-week follow-up into four groups. By analyzing the intergroup difference in indexes and comprehensively comparing these with the results of subproject 1, we assess whether the application of the prediction model is useful for improving treatment efficacy and relieving side effects.

By analyzing the final results, we hope to verify and optimize the prediction model established in subproject 1.

##### To Explore Adjunctive Therapy With Sulforaphane and Minocycline for Patients With Schizophrenia

As is known, negative symptoms are extremely prevalent in the acute episode and long-term course of schizophrenia which are often persistent and tend to lead to chronicity (6). Moreover, cognitive impairment, as a core feature of schizophrenia, begins in the early stages and becomes more severe in the chronic stage of illness (7, 8). However, the evidence for the available psychopharmacological treatments is still not satisfactory, and the antipsychotic drugs appear to show a small benefit in terms of negative symptoms and cognitive impairment (NSACI).

To date, accumulating evidence indicates a role of neuroinflammatory processes in schizophrenia NSACI, and a significant marker of neuroinflammation is microglial activation (9). Minocycline is an antibiotic that has neuroprotective and anti-inflammatory properties, which might prevent or reverse progressive neuropathic changes implicated in recent-onset schizophrenia (10). Our previous research has proven that the inhibition of microglia activation could be one mechanism underlying the antipsychotic effect of minocycline (11). Sulforaphane is a natural compound that is derived from broccoli/broccoli sprouts and is proven to be a potent inducer of cellular antioxidant responses (12). An open study showed that sulforaphane has the potential to improve cognitive function in patients with schizophrenia (13). Accordingly, we conduct a randomized double-blind controlled trial with minocycline and sulforaphane to explore add-on treatment for patients with schizophrenia.

Patients who do not have an ideal response to antipsychotics treatment (reduction rate of Positive and Negative Symptom Scale (PANSS) score <25%) in subproject 2 are recruited in this trial. We perform multiple comparisons of proportions for treatments vs. a control to calculate sample size. We assume that the proportion of successes in the sulforaphane group is 0.3, that in the minocycline group is 0.25, and that in the control group is 0.11 (10, 13, 14). Based on a power of 90%, a significance level of 5%, and 20% of participants lost to follow-up, it was calculated that 227 participants are needed for each group. Therefore, 240 participants are anticipated to be enrolled for each group. They are randomly assigned to antipsychotic plus placebo, antipsychotic plus sulforaphane (3 tables per day, consisting of 30 mg of SFN-glucosinolate per day), and antipsychotic plus minocycline (200 mg per day) groups, and the antipsychotic drugs used at this stage are still consistent with the first trial of subproject 2. The duration of the trial is also 8 weeks. At baseline, 4 weeks, and 8 weeks after treatment, all participants receive the same evaluations as in part 1.

Through an analysis of intergroup differences based on multidimensional evaluation indexes, we will assess the efficacy and safety of minocycline and sulforaphane and further construct an adjunctive therapy for patients with schizophrenia.

Finally, we can optimize and individualize antipsychotic therapy for patients with schizophrenia. Once we generalize the therapy in clinical practice, patients might greatly benefit from it.

### Subproject 3

#### Aim

To construct a comprehensive intervention strategy, which combines medications and lifestyle intervention, based on MetS induced by antipsychotic medications.

We tend to conduct randomized double-blind controlled trials to assess the safety and efficacy of combination strategies for antipsychotic-induced MetS, which includes metformin and lifestyle intervention. Meanwhile, for schizophrenia patients at high risk of MetS, we tend to establish a prevention strategy expected to reduce or delay the occurrence of MetS, which includes low-dose metformin and lifestyle intervention. The setting of the metformin dose is in accordance with that of our previous studies (15–17) and other related studies (18).

For subproject 3, the primary endpoint is the difference in insulin resistance index between groups. According to Wu and Zhao (15), the minimum detectable difference can be assumed as 0.5, with a standard deviation (SD) of 0.5. To detect this difference, based on a power of 90%, a significance level of 5%, and 20% of participants lost to follow-up, it was calculated that 68 participants are needed for each group in the intervention trial, and 62 participants are needed for each group in the prevention trial. The sample size calculation was conducted using Power Analysis and Sample Size statistical software (15.0.5). As a multicenter clinical trial, we set 200 participants for each group.

##### To Establish a Comprehensive Intervention Strategy for MetS Induced by Antipsychotics

Patients with schizophrenia are at an increased risk of developing MetS, which is defined by a combination of abdominal obesity, insulin resistance, dyslipidemia, and elevated blood pressure due to lifestyle- and treatment-related factors (19, 20). All components of MetS have been recognized as independent risk factors for cardiovascular diseases (21). Consequently, it leads to medication discontinuation and poor prognosis for patients with schizophrenia (22).

In our preliminary study, we found that a strategy combining metformin and lifestyle intervention can effectively improve antipsychotic-induced weight gain and insulin resistance (15). Therefore, we plan to carry out a randomized controlled trial to assess the efficacy and safety of this combined intervention in the treatment for antipsychotic-induced MetS.

Participants who develop MetS at the last visit in subproject 1 and subproject 2 are recruited in this trial. The diagnostic criteria are as follows: body mass index ≥25.0 kg/m^2^; hyperglycemia: fasting blood glucose ≥110 mg/dl (6.1 mmol/l) and/or plasma glucose ≥140 mg/dl (7.8 mmol/l) after glucose load and/or those who have been diagnosed with diabetes and received treatment; hypertension: systolic blood pressure/diastolic blood pressure ≥140/90 mmHg and/or those who have been diagnosed with hypertension and received treatment; dyslipidemia: at fasting state, total cholesterol ≥150 mg/dl (1.7 mmol/l) and/or HDL-C for males <35 mg/dl (0.9 mmol/l) and that for females <39 mg/dl (1.0 mmol/l). After signing an ICF, patients are randomized into low-dose metformin (1,000 mg/d), high-dose metformin (1,500 mg/d), low-dose metformin plus lifestyle intervention group (1,000 mg/d), high-dose metformin plus lifestyle intervention (1,500 mg/d), lifestyle intervention, and placebo groups. Lifestyle intervention includes psychoeducational, dietary, and exercise programs as follows: (1) psychoeducational program: we mainly encourage healthy lifestyles and introduce MetS and its lasting bad influences; (2) dietary intervention: the total calories should be controlled below 6,903 kJ/day (proportion: <30% of total calories from fat (<7% saturated fat and <200 mg of cholesterol), 55% from carbohydrates, and more than 15% from protein daily with an increase in fiber intake to at least 15 g per 1,000 kcal; and (3) exercise sessions: participants performed endurance exercise (walking or jogging) on a treadmill seven times per week for 30 min in each session. Exercise is designed to attain 70% of the heart rate reserve [0.7 × (maximum heart rate—resting heart rate) + resting heart rate]. Heart rate reserve is estimated from the maximum and resting heart rates observed at baseline maximal aerobic capacity (Vo_2max_) for each individual. Investigators and patients collaboratively develop individual programs of gradual exercise assignments. A range of different types of exercise and various levels of intensity is offered, varying from light exercise (increased walking and decreased sedentary activities, particularly time spent watching television or sleeping) to moderate exercise for at least 30 min per day. Common moderate-to-vigorous physical exercise includes walking at a moderate or very strenuous intensity, bicycling, resistance training, skiing, jogging, ball games, and lifestyle activities, such as chopping wood or clearing brush (15). We provide a recording sheet, which will be completed by the participant's guardian, to record daily diet and exercises and will follow up on compliance through telephone and internet platforms for participants. The duration of this trial is 12 weeks.

The timepoints of the visits are at baseline, the 4th week, the 8th week, and the 12th week. The detailed information recorded in the case report form (CRF) of each visit is reported in Tables 3, 4.

**Table 3:** Details of follow-up (subproject 3).

**Follow-up**	**1 (baseline)**	**2 (4th week)**	**3 (8th week)**	**4 (12th week)**
Week	0	4	8	12
Written consent	√			
Inclusive/exclusive criterion	√			
Demographic information	√			
Medical history	√			
Physical examination	√	√	√	√
Auxiliary examination	√	√	√	√
Medication compliance	√	√	√	√
Intervention plan	√			
Diet situation	√	√	√	√
Activity situation	√	√	√	√
Collection of blood sample	√		√	√

**Table 4:** Details of laboratory test (subproject 3).

**Follow-up**	**1 (baseline)**	**2 (4th week)**	**3 (8th week)**	**4 (12th week)**
Week	0	4	8	12
Blood regulation	√	√	√	√
Liver function	√	√	√	√
Renal function	√	√	√	√
Blood lipids	√	√	√	√
FBG	√	√	√	√
FI	√	√	√	√
HbA1c	√	√	√	√
OGTT	√	√	√	√
BMI	√	√	√	√
HOMA-IR	√	√	√	√
HBV, HCV	√			
Thyroid function	√			

By analyzing the intergroup difference and intra-group variation in indexes, we will assess the efficacy and safety of different interventions. Finally, we hope to summarize a set of comprehensive intervention strategies for MetS induced by antipsychotic medications.

##### To Establish a Prevention Strategy for Patients With Schizophrenia at a High Risk of MetS

Due to the high prevalence of MetS, it is important to develop and implement prevention strategies for patients with schizophrenia at a high risk, which can improve the occurrence of MetS. Therefore, we plan to carry out a randomized controlled trial to assess the efficacy and safety of drug and non-drug interventions for patients with schizophrenia at a high risk for MetS.

The inclusive criteria are as follows: (1) participants taking antipsychotic drugs that significantly affect metabolism, such as olanzapine, clozapine, or risperidone; (2) participants having a family history of diabetes, hypertension, and heart disease; (3) participants that are over 40 years of age; (4) participants without non-alcoholic fatty liver and gout (23, 24); (5) participants who have one or two components of MetS but do not meet the diagnostic criteria. Participants who are at a high risk of MetS in subproject 1 and subproject 2 are also recruited in this trial after an 8-week treatment.

After completing the ICF, participants are randomized into low-dose metformin (750 mg/d), high-dose metformin (1,000 mg/d), lifestyle intervention, and placebo groups. The purposes and timepoints of every visit are the same as those in part 1 of subproject 3.

After analyzing the intergroup difference and intra-group variation in indexes, we hope to summarize a simple, effective, safe, and inexpensive prevention strategy for patients with schizophrenia at a high risk for MetS.

## Study Evaluation

### Efficacy Evaluation

The primary efficacy evaluation is the total score of PANSS, which is performed by a trained investigator. PANSS is a scale designed to distinguish types and severity of schizophrenia and detect changes in symptoms due to antipsychotic treatment. The PANSS consists of five components in terms of the symptomatology: positive, negative, and cognitive/disorganization symptoms and two other affective dimensions, each of which is scored from 1 (item not present or normal) to 7 (extremely severe presence of symptoms). There is also a semi-structured clinical interview for PANSS (SCI-PANSS) used as a reference for researchers. In the clinical application, PANSS shows great reliability and validity for clinical application during a long period (25–27).

The key secondary efficacy evaluation includes the Global Assessment Function (GAF) and Clinical Global Impression (CGI). The GAF presents the general situation of illness, and patients will be graded from 0 to 100. A lower score indicates worse illness. The CGI is composed of three parts, including severity of illness, global improvement, efficacy index (EI) (EI = treatment efficacy score / adverse effect score). A joint use of three scales makes evaluation a reliable indicator (28).

### Safety Evaluation

Every clinically relevant change occurring during the study must be recorded on the Adverse Event section of the CRF. Moreover, researchers should take corresponding measures to deal with every clinically significant abnormality as follows: (1) vital signs: blood pressure, pulse, respiratory rate, and temperature; (2) physical examination: head and neck, thyroid gland, skin, lymph nodes, heart, lung, abdomen, skeleton and muscle, nervous system, etc.; and (3) EEG: Canadian stellate electroencephalograph (model HSYS-REC-LT2), recording the heart rate, PR interval, QT interval, QRS duration, and examination report from the machine; (4) laboratory tests: blood regulation (white blood cell count, red blood cell count, platelet count, hemoglobin), liver function (aspartate aminotransferase, alanine aminotransferase), renal function (blood urea nitrogen, creatinine, uric acid), fasting blood glucose (FBG), blood lipids (total cholesterol, triglyceride, high-density lipoprotein, and low-density lipoprotein), hepatitis (HBV, HCV), thyroid function (FT3, FT4, TSH), fasting insulin, HbA1c, oral glucose tolerance test; and (5) scales associated with adverse effects: Treatment Emergent Symptom Scale, Systematic Assessment for Treatment Emergent Events, Barnes Akathisia Rating Scale, and A Rating Scale for Extrapyramidal Side Effects.

Adverse event data will be collected by spontaneous self-report and regularly by other evaluations. Participants will be asked to report any problems to their own investigators throughout their participation. We will also ask participants every week if they have any discomfort. The main side effects of antipsychotic treatment are EPS, TD, and MetS. Usually, we administer an antagonist, adjust dosage, or change antipsychotic drugs depending on the severity of side effects. If the situation is life-threatening, we will stop antipsychotic drugs and arrange for participants to be hospitalized as soon as possible. We will conduct an adverse event assessment to determine whether to terminate the study.

### Neurocognitive Assessment

**Figure 2:**
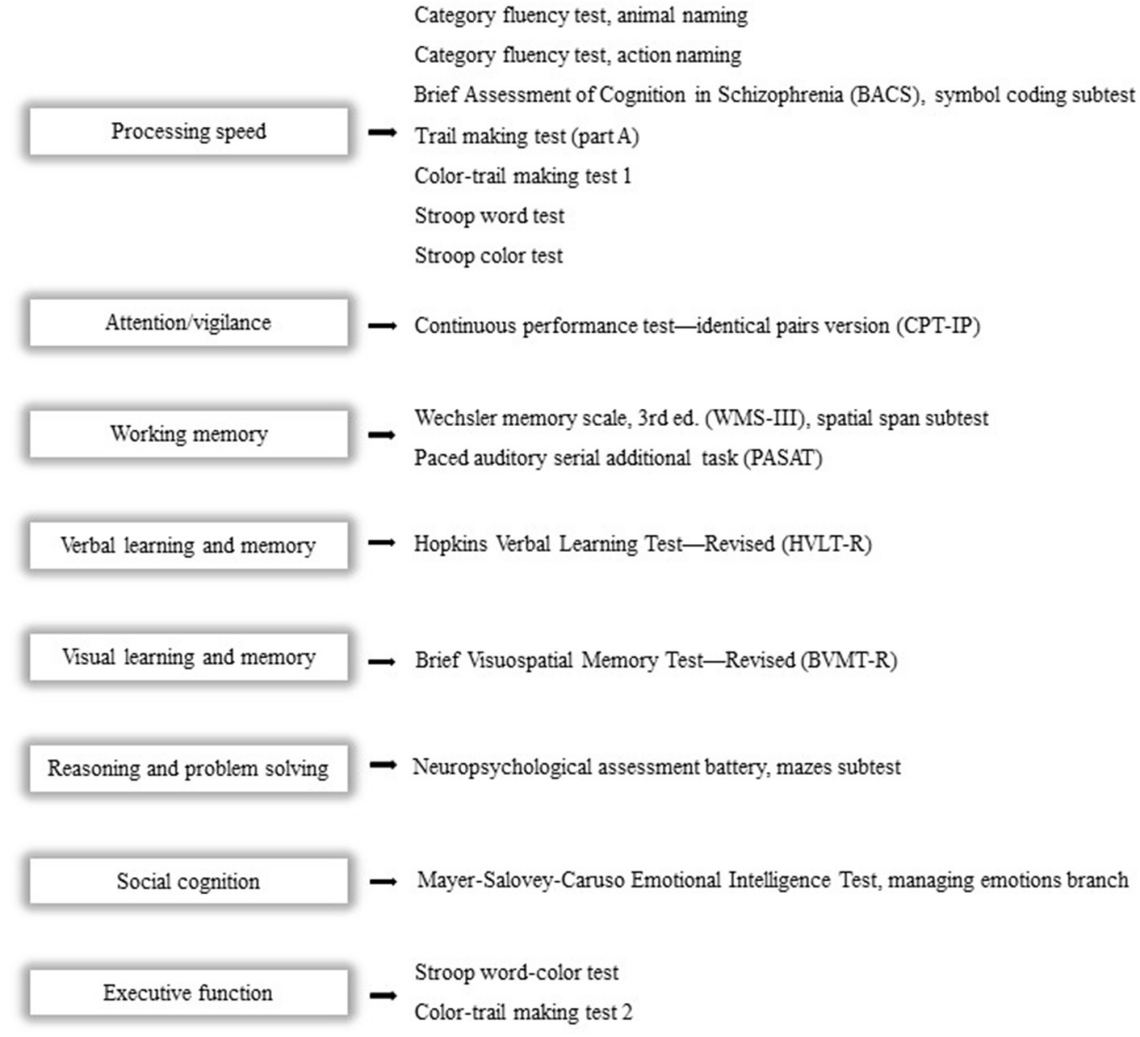
Cognitive domains and corresponding tests. The Chinese version of the MCCB and four supplementary cognitive tests will be used to assess eight cognitive domains as follows: (1) processing speed; (2) attention/vigilance; (3) working memory; (4) verbal learning and memory; (5) visual learning and memory; (6) reasoning and problem solving; (7) social cognition; and (8) executive function.

We use the Chinese version of MATRICS (Measurement and Treatment Research to Improve Cognition in Schizophrenia) Consensus Cognitive Battery (MCCB) and four supplementary cognitive tests to assess eight cognitive domains, which contain the following: (1) processing speed; (2) attention/vigilance; (3) working memory; (4) verbal learning and memory; (5) visual learning and memory; (6) reasoning and problem-solving; (7) social cognition; and (8) executive function (29). The revised and improved Chinese version of MCCB has been widely used as the standard cognitive performance battery for clinical trials of potential cognition-enhancing interventions. After adjusting for age, gender, education years, and region, we convert the raw score into a T-score to generally evaluate cognitive function (30) (Figure 2).

### Measurement of Eye Movement

For participants recruited for this measurement, there are special exclusion criteria: (1) participants' uncorrected visual acuity or corrected visual acuity ≤1.0; (2) participants have a history of ophthalmologic disease or neurological/medical conditions that could influence the central nervous system; (3) participants have a history of atypical headaches, head trauma with a loss of consciousness, epilepsy, seizures, or intellectual disability (IQ ≤ 80); and (4) participants smoke more than 40 cigarettes per day.

The machine for measurement is EyeLink1000 eye movement meter (SR Research, Ontario, Canada; 500 Hz). The method for determination is as follows: the display monitor is placed at a distance of 70 cm from the observers' eyes, and the light source is set up at 75 or 100% according to the surrounding light conditions. During the tasks, we track single eye movement through a 30-mm lens. Moreover, the following procedures include a nine-point map calibration, verification calibration, and offset correction during the entire measurement. The stimulation tasks are presented in turn as follows: (1) free view, (2) smooth pursuit, (3) fixation stability, (4) prosaccade, and (5) anti-saccade.

### MRI Examination

Every participant undergoes structural MRI scanned through a high-resolution 3-dimensional T1-weighted sequence scan with a spoiled gradient recalled sequence and the resting-state functional MRI scanned with a gradient-echo echo-planar imaging sequence. The evaluation indexes include items such as cerebral gray matter volume, neuronal activity regional homogeneity, fractional amplitude of low-frequency fluctuations, network homogeneity, and functional connectivity.

### Measurement of Pharmacogenomics

#### Verification of Known Genetic Markers Associated With Antipsychotics

Combined with the databases of Thousand Genome, NCBI, ExAC, and HDGP, we screen the loci that are of instructive significance for the Chinese population. Then, based on the latest research on these loci, we capture and sequence the target genes of participants' DNA, of which 106 mutations are known to affect the efficacy of drugs. Finally, we determine the correlation between the sequence of target genes and clinical outcomes for verification.

#### Discovery of New Genetic Targets Associated With Antipsychotics

In each antipsychotic monotherapy group, we select 50 responsive participants and 50 non-responsive participants. Through high-throughput methods, such as second-generation sequencing, we obtain genomic information for them. Then, we divide the 50 patients into two groups according to the extent of the severity of adverse effects. By combining this with clinical phenotypes, we use bioinformatics methods to locate new targets related to drug efficacy and adverse effects.

#### Research on Clarifying the Mechanism of Action of New Genetic Mutations

Among the newly found mutations, we choose those that remarkably influence metabolism and treatment response. In the next animal experiment, we construct mouse models using the CRISPR/Cas9 method, which are next induced to develop psychosis by phencyclidine and evaluate if the psychosis model is successful by observing the behaviors of mice. There are three groups, including the model group, control group, and model treatment group. Moreover, we assess the treatment efficacy through behavioral observations, the influence of mutations on efficacy or toxicity, and potential mechanism, which will be studied by pharmacokinetics and pharmacodynamics.

#### To Establish a Genetic Biomarker Model for Predicting Antipsychotic Response

First, we screen genetic markers as mentioned previously, including known and newly found ones that are strongly correlated with response. Then, the aim will be to establish a prediction model and further to verify it with the original and test samples.

### Other Evaluation Indexes

Epigenetic indexes will include the following: DNA methylation (DNMT, GADD67) and the expression levels of miRNA and protein of DNA demethylation-associated markers (APOBEC3A/3C, MBD4). Neuroimmunological indexes will include the following: interleukin-1 β (IL-1 β), interleukin-6 (IL-6), and tumor necrosis factor-α (TNF-α). Metabolomics indexes will include the following: lipid metabolites, amino acid metabolites, carbohydrate metabolites, and nucleotide metabolites. Metabolic signs will include weight, height, waist circumference, and hip circumference.

## Ethics and Dissemination

The study protocol has been approved by the Medical Ethics committee of the Second Xiangya Hospital of Central South University (No. 2017027). All substantial amendments of this protocol will be submitted to the Medical Ethics committee for review.

ICFs will be filled out by the patients and their legal representatives. Before inclusion, the investigator or an authorized member of the study will explain the purpose, the methods, privacy protection, reasonably anticipated benefits, and potential hazards of the study to the potential subjects and their legal representatives. Subjects will be informed that the study is completely based on the principle of voluntariness and that they have the right to cease participation at any time. Regardless of their choice, the treatment of their disease will not be influenced. The investigator will give sufficient time for the subject to assess every detail and make a decision.

The results of this study will be disseminated via an international peer-reviewed publication. Further, it will be presented at national and international conferences as posters or oral presentations for communication and discussion.

## Project Funding and Infrastructure

This project is funded by the National Key Research and Development Program (2016YFC1306900). As a national clinical research center for mental disorders, the psychiatry department of the Second Xiangya Hospital, Central South University, provides a mature laboratory environment and large advantages regarding cooperating with experts in various fields. Experts from the other 19 hospitals around the country are also part of this project to explore optimized and individualized therapy for patients with schizophrenia.

All experts and researchers met together for an initial project meeting to discuss the overall study goal and research procedure. Moreover, we conducted unified training for researchers of each hospital so that they can perform evaluations consistently. Further, we will communicate with cooperators and examine the blood samples and CRFs to resolve difficulties and correct errors in a timely manner.

## Summary

This project was initiated in September 2016 and was expected to complete enrollment for the three subprojects before December 31, 2020. However, due to the Coronavirus disease (COVID-19) pandemic, the deadline is estimated to be postponed about 1 year. By investigating an optimized and individualized antipsychotic treatment for schizophrenia patients in this project, we can, hopefully, improve the treatment for schizophrenia and its prognosis.

## Ethics Statement

The studies involving human participants were reviewed and approved by the Medical Ethics committee of the Second Xiangya Hospital of Central South University. Written informed consent to participate in this study was provided by the participants' legal guardian/next of kin.

## Author Contributions

JX, YL, XW, YW, YY, GH, MS, JinZ, LL, TS, WW, DK, CL, PX, and YH are involved in participant recruitment, follow-up evaluation, and data and sample collection. JingZ and RW are involved in study design, protocol preparation, acquisition of funding, and responsible for the project concept. JX and JH are responsible for the trial protocol draft and final revision. All authors have reviewed and provided critical revision of the manuscript.

## Conflict of Interest

The authors declare that the research was conducted in the absence of any commercial or financial relationships that could be construed as a potential conflict of interest.
